# Characteristics of human papillomavirus infection among females and the genetic variations of HPV18 and HPV58 in Henan province, China

**DOI:** 10.1038/s41598-022-24641-4

**Published:** 2023-02-08

**Authors:** Ying Wang, Shuizhong Han, Xiaochuan Wang, Shuya Song, Xiuli Wang

**Affiliations:** 1 College of Food and Drugs, Luoyang Polytechnic, Luoyang, Henan China; 2 College of Veterinary Medicine, Northwest A&F University, Yangling, Shaanxi China; 3 Department of Blood Transfusion and Clinical Laboratory, No.989 Hospital of the Joint Logistic Support Force of Chinese PLA, Luoyang, Henan China

**Keywords:** Cancer, Genetics, Microbiology

## Abstract

The present study aims to investigate the genotype distribution of Human papillomavirus (HPV) and variations of HPV18 and HPV58 infection among 6538 females in Luoyang city during 2019–2021. The overall positive rate of females with HPV infection was 12.34%, with 9.74% were infected with single HPV and 2.60% with multiple HPVs. The prevalent rate of high-risk HPV (HR-HPV) was 9.85% and the top five HR-HPV genotypes were HPV52 (1.94%), HPV16 (1.93%), HPV58 (1.48%), HPV51 (1.02%) and HPVV39 (0.99%). Two peaks of HPV infections rates were observed in females aged ≤ 20 and 61–65 years old. To characterize mutations, 39 HPV18 and 56 HPV58 L1, E6 and E7 genes were sequenced and submitted to GenBank. In the HPV18 E6-E7-L1 sequences, 38 nucleotides changes were observed with 10/38 were non-synonymous mutations (5 in E6 gene, 1 in E7 gene and 4 in L1 gene). In the HPV58 E6-E7-L1 sequences, 53 nucleotides changes were observed with 23/53 were non-synonymous mutations (3 in E6 gene, 5 in E7 gene and 15 in L1 gene). Phylogenetic analysis based on L1 gene showed that 92.3% (36/39) of HPV18 isolates fell into sublineage A1 and 7.7% (3/39) belonged to A5. For HPV58, 75.0% (42/56) isolates belonged to sublineage A1 and 25.0% (14/56) were sublineage A2. There was no association between amino mutation and cervical lesions. The present study provides basic information about the distribution, genotypes and variations of HPV among females population in Luoyang city, which would assist in the formulation of HPV screening and vaccination programs and preventive strategies for HPV-attributable cancer in this region.

## Introduction

Globally, it was estimated that there were 604,000 cases and 342,000 deaths were caused by cervical cancer in 2020, which ranked the fourth most frequently diagnosed cancers^1^. Females lived in developing countries contributed about 85% cervical cancer and the death rate was higher than developed countries (12.4 vs 5.2 per 100,000)^1,2^. In 2022, there would be approximately 111,820 new cases of cervical cancer and 61,579 cancer deaths in China^3^.

Human papillomavirus (HPV) are identified in most patients with cervical carcinoma and the persistent infection with one or more genotypes of oncogenic HPV are the necessary causes of cervical cancer^4–6^. HPV is a small circular non-enveloped double-stranded DNA virus that belongs to the family Papillomavirus^7^. There are more than 200 different HPVs genotypes have been identified and classified into high-risk HPV (HR-HPV) and low-risk HPV (LR-HPV) based on their potential to cause cancer^8^. Globally, the most common HPV genotypes in invasive cervical cancer were 16, 18, 31, 33, 35, 45, 52 and 58^9^. Meanwhile, it was reported that the distribution of HPV genotypes exhibited significant differences among countries, even in different areas of a country. For example, the top prevalent HR-HPV genotype was HPV52 in Beijing city and HPV16 in Shanghai city of China^10,11^. The preventive effect of HPV vaccines on cervical cancer and other HPV-associated diseases has been confirmed in multiple studies^12,13^. Currently, commercial 2-valent (HPV16 and HPV18), 4-valent (HPV 6, 11, 16 and 18) and 9-valent (HPV 6, 11, 16, 18, 31, 33, 45, 52 and 58) HPV vaccines have been approved by the National Medical Products Administration of China. Some provinces of China, such as Guangdong, have piloted the HPV vaccination program among the native females. Understanding the distribution of HPV genotypes in a region will provide baseline information for the implementation of vaccine-based HPV prevention strategies. Until now, there is limited information about the genotype distribution of HPV in Henan province, located in central of China.

Epidemiological study had showed that except HPV16, HPV18 and HPV58 were the most prevalence genotypes among females with cervical lesions in Henan province of China^14^. HPV18 can be divided into three lineages and ten sublineages: (1) A, A1–A6; (2) B, B1, B2 and B3; (3) C^15^. HPV58 has been classified into four lineages and eight sublineages: (1) A, A1–A3; (2) B, B1 and B2; (3) C; (4) D, D1 and D2^15^. The sublineage of HPV18 and HPV58 had been determined in Hunan and Zhejiang province of China and the predominant sublineage was determined to be sublineage A1^16,17^. To our knowledge, there are no reports on the sublineages and sequence variations of HPV18 and HPV58 in Henan province.

Object of the present study is to investigate the genotypes distribution and variations of HPV18 and HPV58 among females in Luoyang city, Henan province, located in central China. The investigation would assist on the formulation and development of vaccine-based HPV prevention strategies against cervical cancer.

## Results

### Characteristics of the study participants

A total of 6538 females were included in this study and 807 (12.34%) were infected with HPV. As shown in Table 1, the positive rate of HR-HPV was 9.85%, higher than LR-HPV (3.79%) (*χ*^2^ = 188.673, *P* < 0.01). The top five HR-HPV genotypes were HPV52 (1.94%), HPV16 (1.93%), HPV58 (1.48%), HPV51 (1.02%) and HPV39 (0.99%). The most prevalent LR-HPV genotypes were HPV61 (0.89%), followed by HPV54 (0.72%), HPV81 (0.60%), HPV6 (0.37%) and HPV11 (0.29%). Among the 807 HPV positive females, 637 were infected with single HPV genotype, 170 were infected with more than one HPV genotype. Among the 637 females infected with single HPV genotype, the HR-HPV infection accounted for 76.45% (487/637). The multiple HR-HPV infection rate was 2.40%, higher than the multiple LR-HPV infection (1.50%) (*χ*^2^ = 13.922, *P* < 0.01). The top prevalent of the multiple HPV infection was HPV52 (0.70%), followed by HPV58 (0.55%), HPV16 (0.47%), HPV39 (0.41%) and HPV51 (0.41%).

**Table 1:** The prevalence of 37 HPV genotypes of single/multiple infection in all the specimens (n = 6538).

Category	Numbers	Overall prevalence (100%)	Single infection		Multiple infection	
HPV type			Numbers	Prevalence	Numbers	Prevalence
Any HPV type	807	12.34%	637	9.74%	170	2.60%
Low risk types	248	3.79%	150	2.29%	98	1.50%
6	24	0.37%	11	0.17%	13	0.20%
11	19	0.29%	15	0.23%	4	0.06%
34	16	0.24%	8	0.12%	8	0.12%
40	6	0.09%	3	0.05%	3	0.05%
42	18	0.28%	11	0.17%	7	0.11%
43	4	0.06%	2	0.03%	2	0.03%
44	3	0.05%	2	0.03%	1	0.02%
54	47	0.72%	26	0.40%	21	0.32%
55	7	0.11%	0	0.00%	7	0.11%
57	0	0.00%	0	0.00%	0	0.00%
61	58	0.89%	34	0.52%	24	0.37%
67	3	0.05%	2	0.03%	1	0.02%
69	4	0.06%	3	0.05%	1	0.02%
70	5	0.08%	1	0.02%	4	0.06%
71	1	0.02%	0	0.00%	1	0.02%
72	0	0.00%	0	0.00%	0	0.00%
81	39	0.60%	22	0.34%	17	0.26%
83	4	0.06%	2	0.03%	2	0.03%
84	18	0.28%	8	0.12%	10	0.15%
High risk types	644	9.85%	487	7.45%	157	2.40%
16	126	1.93%	95	1.45%	31	0.47%
18	63	0.96%	39	0.60%	24	0.37%
26	0	0.00%	0	0.00%	0	0.00%
31	23	0.35%	14	0.21%	9	0.14%
33	36	0.55%	24	0.37%	12	0.18%
35	13	0.20%	9	0.14%	4	0.06%
39	65	0.99%	38	0.58%	27	0.41%
45	7	0.11%	3	0.05%	4	0.06%
51	67	1.02%	40	0.61%	27	0.41%
52	127	1.94%	81	1.24%	46	0.70%
53	28	0.43%	13	0.20%	15	0.23%
56	22	0.34%	12	0.18%	10	0.15%
58	97	1.48%	61	0.93%	36	0.55%
59	12	0.18%	4	0.06%	8	0.12%
66	54	0.83%	32	0.49%	22	0.34%
68	41	0.63%	22	0.34%	19	0.29%
73	1	0.02%	0	0.00%	1	0.02%
82	0	0.00%	0	0.00%	0	0.00%

### Prevalence of HPV infection in different age groups

**Figure 1:**
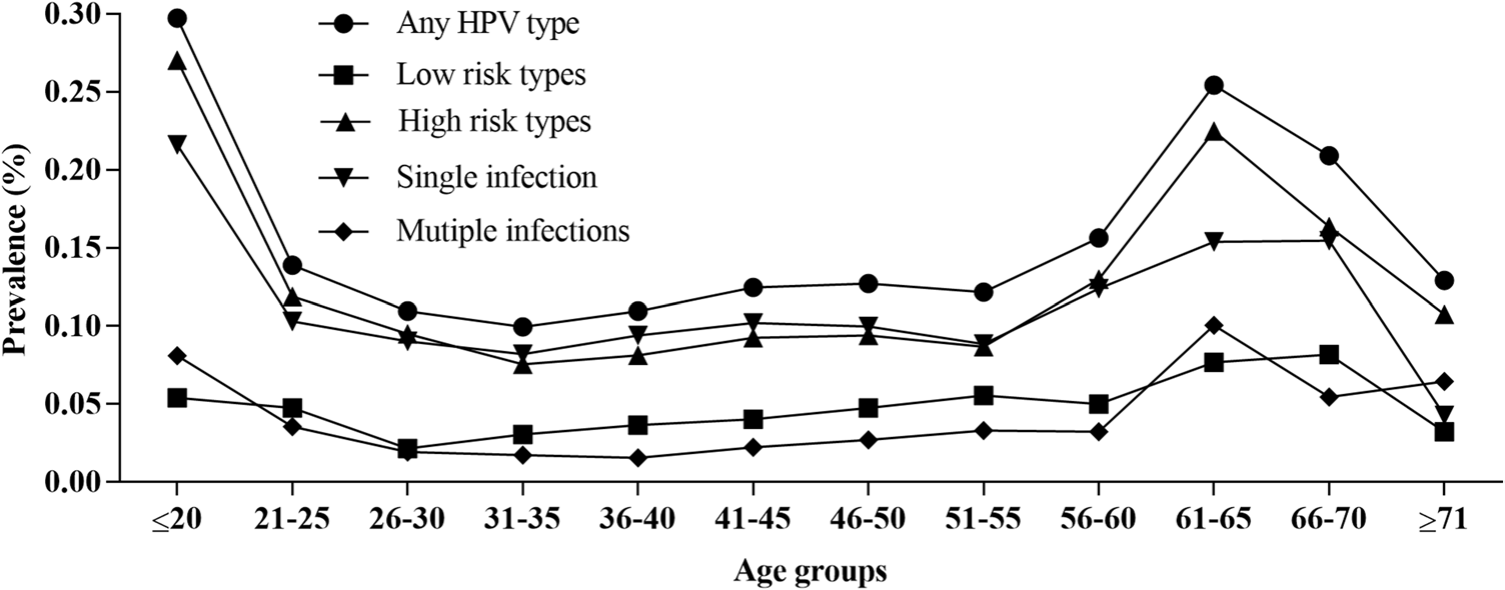
Prevalence of the HPV infection types in different age groups.

There are significant differences in the positive rates of HPV infection in different age groups (*χ*^2^ = 149.128, *P* < 0.01). The highest rate of any HPV type infection was observed in the ≤ 20 year-old group (29.73%, 11/37), followed by 61–65 year-old group (25.44%, 43/169), while the 31–35 year-old group had the lowest prevalence rate (9.95%, 120/1206). As shown in Fig. 1, the LR-HPV, HR-HPV, single and multiple infection groups showed an identical tendency to the “Any HPV type” infection in different age groups.

### Variations of L1 genes on HPV18 and HPV58

Thirty-nine HPV18 and fifty-six HPV58 L1 genes were sequenced successfully. Except for the same sequences, thirteen different HPV18 (18HNL01-18HNL13) and twenty-four HPV58 (58HNL01-58HNL24) sequences were submitted to GenBank (the accession numbers are OP684028-OP684040 and OP684041-OP684064). The HPV18 (AY262282) and HPV58 (D90400) reference sequences were used as the standard for comparison and position of the gene polymorphism sites, separately. The nucleotide sequence mutations of the studied sequences were shown in Tables 2 and 3.

**Table 2:** Nucleotide sequence mutations of HPV18 L1 gene.

nt	Domain: HPV18 L1 sequence																					Numbers	Sublineages
	5	5	5	5	5	5	5	5	5	5	5	5	5	6	6	6	6	6	6	7	7		
	4	5	5	6	7	7	8	8	8	9	9	9	9	0	0	1	1	4	4	0	1		
	7	0	7	7	4	9	0	3	7	2	2	5	9	7	8	4	7	0	0	7	3		
	4	3	0	1	1	6	1	2	5	0	4	2	1	1	9	3	0	0	6	9	0		
AY262282	A	G	T	G	A	A	T	A	C	C	A	G	G	G	G	G	C	G	A	A	G		Protype
18HNL01		A								T												3	A1
18HNL02		A																				19	A1
18HNL03	G	A			G	G			A						A	C			G	T	A	3	A5
18HNL04		A																				1	A1
18HNL05		A	G																			1	A1
18HNL06		A												A								1	A1
18HNL07		A								T												1	A1
18HNL08		A					C											A				1	A1
18HNL09		A								T								A				1	A1
18HNL10		A										A	C									1	A1
18HNL11		A						C			C											2	A1
18HNL12		A						C			C						A					4	A1
18HNL13		A		A						T												1	A1
Reference aa		R				I			T	A													
aa position						1			1	1													
		2				2			4	6													
		5				3			9	4													
aa mutations		Q				V			N	V													
Frequency	3	39	1	4	3	3	1	6	3	5	6	1	1	1	3	3	4	2	3	3	3	39	

Positions without variants are marked with blank, whereas positions with variants are indicated by a letter.

**Table 3:** Nucleotide sequence mutations of HPV58 L1 genes.

nt	5	5	5	5	5	5	6	6	6	6	6	6	6	6	6	6	6	6	6	6	6	6	6	6	6	6	6	6	6	6	6	7	7	7	Numbers	Sublineages
	7	8	8	8	8	9	0	0	0	0	3	3	4	4	4	4	4	4	5	5	5	5	5	5	6	6	7	7	7	8	6	0	0	1		
	0	0	2	3	4	6	1	1	5	6	9	9	1	1	2	3	4	5	1	1	2	3	5	6	4	8	0	4	9	2	9	0	3	3		
	2	9	9	4	3	1	0	4	6	2	0	1	5	6	6	4	1	8	2	7	1	9	4	0	1	8	6	6	7	7	0	0	6	2		
D90400	G	A	A	C	A	G	A	A	A	A	C	T	G	A	A	T	C	G	A	T	A	A	A	A	G	C	A	A	G	C	A	A	G	A		Protype
58HNL01		C		A				C												A						A									1	A2
58HNL02																								G		A									12	A1
58HNL03																								G		A									1	A1
58HNL04								C						G		C						G			A										6	A2
58HNL05								C						G		C		A				G			A										1	A2
58HNL06			G					C						G		C			G		G	G			A										1	A2
58HNL07																															G				5	A1
58HNL08								C						G		C						G			C										1	A2
58HNL09						A																											C		1	A1
58HNL10																	A																		4	A1
58HNL11	A				C																			G										C	1	A1
58HNL12																								G		A			A			C			4	A1
58HNL13										G														G		A									4	A1
58HNL14								C						G								G			A		C								1	A2
58HNL15										G																									1	A1
58HNL16											T	C	A		T									G		A									1	A1
58HNL17									G																										3	A1
58HNL18								C						G		C						G			A										1	A2
58HNL19								C						G		C						G			A										2	A2
58HNL20																								G											1	A1
58HNL21																							G	G											1	A1
58HNL22								C						G		C						G		G	A					T					1	A2
58HNL23																								G		A									1	A1
58HNL24							G																					G							1	A1
Reference aa		N				E	Y	L			L		R		N		L			F		I				T	K					K	R	K		
aa position						1	1	1			2		2		2		2			3		3				3	3					4	4	5		
		8				3	4	5			7		8		8		9			1		2				7	8					7	9	2		
		2				3	9	0			6		4		8		3			8		5				5	1					9	1	3		
aa mutations		T				K	C	F			S		K		Y		I			Y		M				N	T					T	P	T		
Frenquency	1	1	1	1	1	1	1	15	3	5	1	1	1	14	1	13	4	1	1	1	1	14	1	27	14	24	1	1	4	1	5	4	1	1	56	

Positions without variants are marked with blank, whereas positions with variants are indicated by a letter.

For HPV18 L1 gene, 18HNL02 sequence accounted for 48.7% (19/39) and was the predominant strain. Twenty-one variations were observed in HPV18 L1 gene and four were non-synonymous mutations, including G5503A (R25Q, 39/39), C5920T (A164V, 5/39), A5796G (I123V, 3/39) and C5875A (T149N, 3/39). The most frequency synonymous mutations were A5832C (6/39) and A5924C (6/39) in HPV18 L1 gene.

For HPV58 L1 gene, fifteen nucleotide changes were non-synonymous mutations. The most prevalence synonymous variation was A6560G (27/56), which was found in 26 HPV58 sublineage A1 isolates. The highest rate of non-synonymous mutations was C6688A (T375N, 24/56) and was observed in 24 HPV58 sublineage A1 isolates. The T6434C, A6539G and G6641A variations were only found in sublineage A2. The A6014C synonymous variation was found in 15 HPV58 sequences and 14 were sublineage A2.

### Variations of E6–E7 genes on HPV18 and HPV58

A total of thirty-nine HPV18 and fifty-six E6–E7 genes were gained and the nucleotide and amino acid sequences variations are summarized in Tables 4 and 5. For HPV18, thirteen different E6-E7 gene sequences (18HNE01–18HNE13) were submitted to GenBank (the accession numbers are OP684065–OP684077 for HPV18 E6, OP684078–OP684090 for HPV18 E7). The 18HNE01 represented the most predominant strain (27/39), which shared the same sequence with the HPV18 reference strain (AY262282). Five non-synonymous and four synonymous variations were identified on E6 gene. One non-synonymous and seven synonymous were observed on E7 gene. The non-synonymous mutations on E6 gene were E29Q, E40K, R74K and L93R; and on E7 was Q222H.

**Table 4:** Nucleotide sequence mutations of HPV18 E6-E7 genes.

nt	Domain: HPV18 E6–E7 sequence																	Numbers
	1	1	1	2	2	3	3	3	4	4	5	5	5	6	7	8	8	
	4	5	8	1	2	2	7	8	5	8	4	5	9	4	7	6	6	
	9	3	9	8	2	5	7	2	2	5	9	1	3	0	0	0	4	
AY262282	T	C	G	A	G	G	A	T	A	T	C	A	C	C	A	C	A	
18HNE01																		27
18HNE02		T					G			C	A				C			1
18HNE03						A												1
18HNE04										C								1
18HNE05								G										1
18HNE06										C	A							1
18HNE07				T						C	A							1
18HNE08																T		1
18HNE09	C																	1
18HNE10									C									1
18HNE11			C															1
18HNE12					A													1
18HNE13												C	T	T			G	1
Reference aa			E		E	R		L							Q			
aa position															2			
			2		4	7		9							2			
			9		0	4		3							2			
aa mutations			Q		K	K		R						T	H			
Frequency	1	1	1	1	1	1	1	1	1	4	3	1	1	1	1	1	1	39

Positions without variants are marked with blank, whereas positions with variants are indicated by a letter.

**Table 5:** Nucleotide sequence mutations of HPV58 E6-E7 genes.

nt	Domain: HPV58 E6–E7 sequence													Numbers
	2	3	3	3	3	5	6	6	7	7	7	7	8	
	5	0	2	7	8	9	3	9	2	4	6	6	0	
	9	7	1	4	8	9	2	4	6	4	0	1	3	
D90400	A	C	C	G	A	G	C	G	T	T	G	G	T	
58HNE01									C					3
58HNE02					C					G			C	16
58HNE03		T						A		G		A		4
58HNE04														6
58HNE05		T					T			G	A			4
58HNE06	G									G			C	5
58HNE07					C									5
58HNE08				A	C					G			C	3
58HNE09		T						A		G		A	C	3
58HNE10			T		C					G			C	4
58HNE11					C	A				G			C	3
Reference aa			S	E	K	R	T	G			G	G	V	
aa position			7	8	9		2	4			6	6	7	
			1	9	3	9	0	1			3	3	7	
aa mutations			F	K	N	K	I	R			S	D	A	
Frequency	5	11	4	3	31	3	4	7	3	42	4	7	34	56

Positions without variants are marked with blank, whereas positions with variants are indicated by a letter.

For HPV58, eleven different E6-E7 sequences (58HNE01–58HNE11) were gained and submitted to GenBank (the accession numbers are OP684091-OP684101 for HPV58 E6, OP684017-OP684027 for HPV58 E7). A total of thirteen gene mutations were observed and five were on E6 and eight on E7. The most frequency synonymous mutation was C307T (11/56) in E6 gene and T744G (42/56) in E7 gene. The most prevalent non-synonymous mutations were A388C (K93N) on E6 gene and T803C (V77A) on E7 gene.

### Phylogenetic analysis

**Figure 2:**
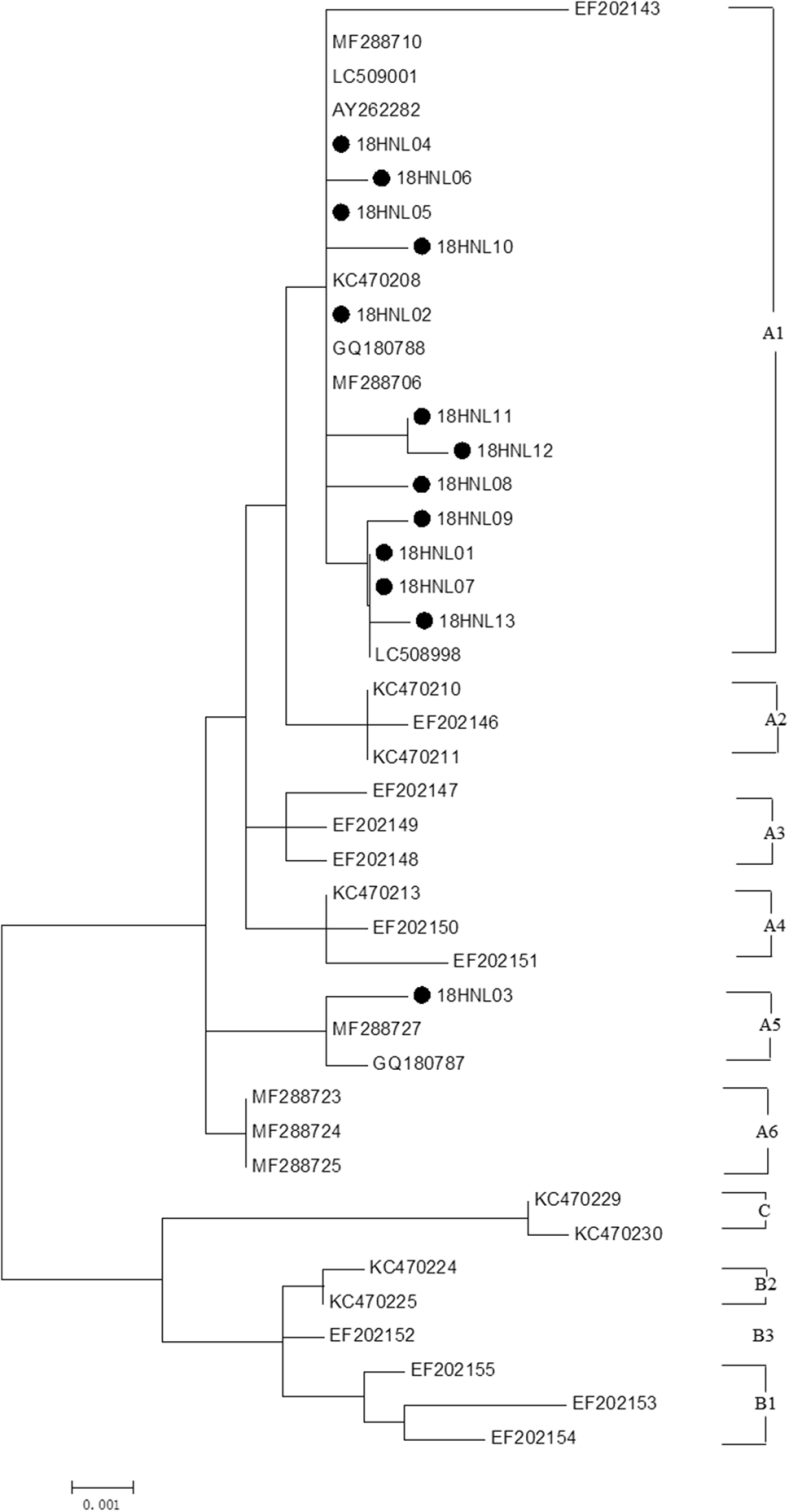
Phylogenetic tree generated using nucleotide sequences of the HPV18 L1 gene. Study sequences are labeled in dots, others without dots are reference strain, including: A1 (AY262282, EF202143, MF288710, LC509001, KC470208, GQ180788, MF288706, LC508998), A2 (EF202146, KC470210, KC470211), A3 (EF202147, EF202148, EF202149), A4 (EF202150, EF202151, KC470213), A5 (GQ180787, MF288727), A6 (MF288723, MF288724, MF288725), B1 (EF202153, EF202154, EF202155), B2 (KC470224, KC470225), B3 (EF202152) and C (KC470229, KC470230). Phylogenetic trees were constructed by the Maximum Likelihood by MEGA 6.0 package. Only bootstrap values above 70% are displayed in the branches.

**Figure 3:**
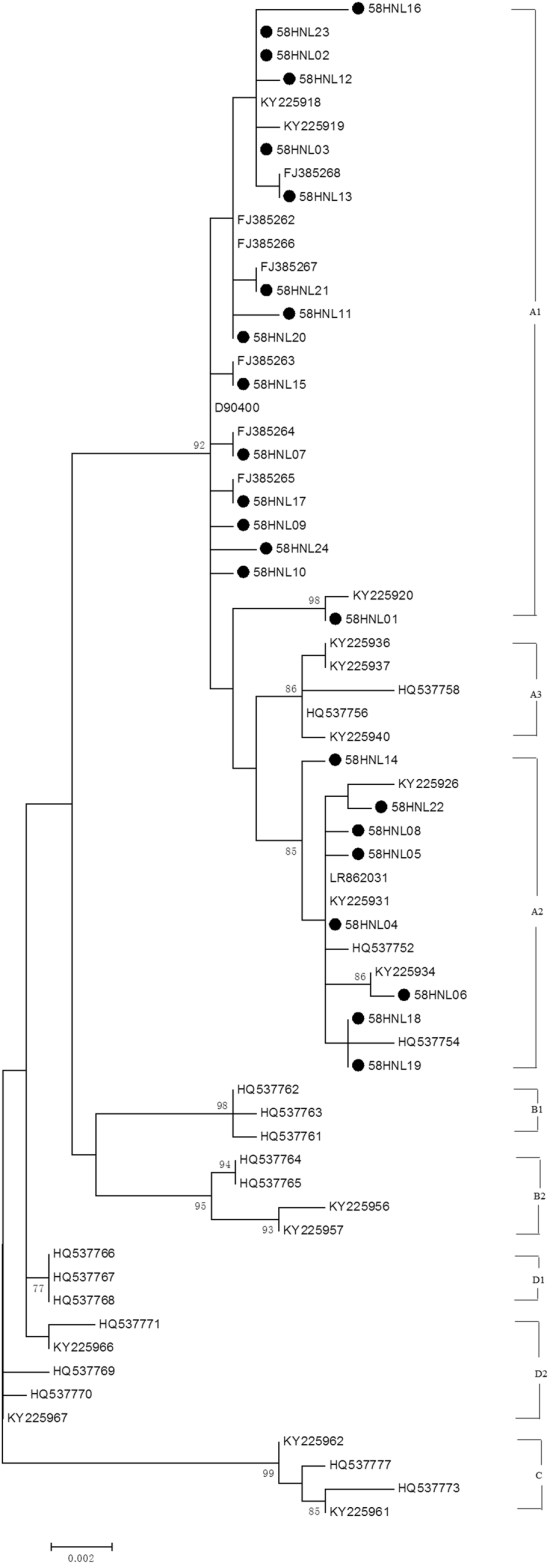
Phylogenetic tree generated using nucleotide sequences of the HPV58 L1 gene. Study sequences are labeled in dots, others without dots are reference strain, including: A1 (D90400, KY225918, KY225919, FJ385262-FJ385268), A2 (KY225926, KY225931, KY225934, HQ537752), A3 (KY225936, KY225937, KY225940, HQ537756, HQ537758), B1 (HQ537761-HQ537763), B2 (HQ537764, HQ537765, KY225956, KY225957), C (KY225961, KY225962, HQ537773, HQ537777), D1 (HQ537766-HQ537768) and D2 (KY225966, KY225967, HQ537768-HQ537770). Phylogenetic trees were constructed by the Maximum Likelihood method by MEGA 6.0 package. Only bootstrap values above 70% are displayed in the branches.

Phylogenetic trees based on the full length of HPV18/58 L1 genes were constructed, together with those of reference HPV18/58 L1 sequences that represent individual variant lineages/sublineages. As shown in Fig. 2, 92.3% (36/39) of HPV18 isolates fell into sublineage A1 and 7.7% (3/39) belonged to sublineage A5. Among the fifty-six HPV58 isolates, 75.0% (42/56) belonged to sublineage A1 and 25.0% (14/56) were sublineage A2 (Fig. 3).

### Risk association with cervical lesions

As each non-synonymous mutation in HPV18 E6–E7 gene has only one sample, thus, the risk association of amino mutation with cervical lesions was estimated on HPV58 only. Among the fifty-six females infected only with HPV58, thirty-one were diagnosed with normal cervix by colposcopy examination and twenty-five were diagnosed with CIN2 or worse. It showed that there was no association between amino mutation and cervical lesions (Table 6).

**Table 6:** Analysis on the oncogenic risk association of HPV58 E6 and E7 amino substitutions.

Gene	Amino acid substitution	Cervical pathology		*P*-value	Odds ratio (95% CI)
		Normal cervices(N = 31)	CIN2 + (N = 25)		
HPV58 E6	S71F	1	2	0.581	2.069(0.223–30.569)
	E89K	2	1	1.000	0.604(0.052–7.076)
	K93N	15	16	0.288	1.896(0.645–5.575)
HPV58 E7	R9K	1	2	0.581	2.069(0.223–30.569)
	T20I	2	2	1.000	1.261(0.165–9.648)
	G41R	4	3	1.000	0.920(0.186–4.556)
	G63S	2	2	1.000	1.261(0.165–9.648)
	G63D	4	3	1.000	0.920(0.186–4.556)
	V77A	19	15	1.000	0.947(0.322–2.785)

## Discussion

Cervical cancer is the leading cause of deaths in China and it was estimated that there were 111,820 new cases and 61,579 deaths in Chinese females in 2022^3^. Persist infection with HR-HPV is known to be the necessary causes of cervical cancer^4–6^. A retrospective study showed that HPV16 and HPV18 were detected in 71% invasive cervical cancer in the world^9^. In the present study, the distribution of HPV genotypes among 6538 females who underwent gynecological outpatient clinic during 2019–2021 was investigated in Luoyang city. It showed that the overall prevalence of HPV was 12.34%, which was similar to Zhengzhou city of Henan province, but lower than Beijing (21.06%) or Shanghai (18.98%) city^10,11,18^. The most prevalent HR-HPV genotypes in Luoyang city were HPV52, 16, 58, 51 and 39. The 2-valent, 4-valent and 9-valent HPV vaccines cover 28.6%, 34.0% and 73.1% of HR-HPV positive samples in the present study (data not show). Vaccines contained HPV51 and HPV39 would cover 87.6% of HR-HPV infection in Luoyang city (data not show), which should be taken into consideration in future. In China, the knowledge score and proportion of females who were willing to receive HPV vaccine were relative low^19^. More efforts should be made by the government to increase the awareness and knowledge of HPV vaccine.

The prevalence of HPV infection in different age population was calculated. It showed that there were two peaks among females with HPV infections, one was ≤ 20, and the other was 61–65 year-old females. The two peaks of HPV infection age group were also observed in other reports^18,20–22^. The first peak of HPV infection may be due to the lack of immunity to HPV in ≤ 20 year-old females^23^. Thus, the adolescent girls should take priority in the HPV vaccine program. The second peak occurred within the age group 61–65, which was assumed to be caused by the physiologic and immunologic deregulation^24^. In China, though the government has made enormous investment on cancer screen since 2009, more attention should be paid for females around 60 years old^25^.

Globally, HPV18 is the second most carcinogenic HPV genotype and has a higher proportion in cervical adenocarcinomas (ADC)^26^. HPV58 accounted for 6.4% in invasive cervical cancer worldwide, which was especially higher in Eastern Asia^27,28^. It was reported that HPV18 and HPV58 had a significant association with an increased risk for cervical cancer in China^14,29–31^. Genetic variations and sublineage of HPV may affect the pathogenic potential and host immune responses^32–35^. In the present study, the L1, E6 and E7 gene sequences of HPV18 and HPV58 were sequenced. Phylogenetic tree based on the L1 genes showed that the most common HPV18 sublineage was A1, which was similar to other provinces of China^16,17^. In other countries in Eastern Asia and Pacific, such as Korea and Japan, the predominant HPV18 sublineage was also A1^36,37^. Compared with HPV18 lineage A, the HPV18 B/C tend to cause higher cancer risks^38^. Four non-synonymous mutations were found in HPV18 L1 gene, including R25Q, I123V, T149N and A164V, which had been detected in Zhejiang province of China^17^. The R25Q, T149N and A164V mutations were also prevalent in Korea HPV18 sublineage A1 isolates^36^. In China, it was reported that the distribution of R25Q mutation differed with geographical region and racial characteristic^36,39^. The A5474G, A5741G, A5796G, C5875A, G6089A, G6143C, A6406G, A7079T and G7130A were only found in HNL03, which represented sublineage A5. Compared with the reference HPV18 sublineage A5 (Accession number: GQ180787), A5468G, A5790G, T5914C, A7073T substitutions were found in L1 sequence. For HPV18 E6 and E7 gene, there were twenty-seven HNE01 isolates that shared the same sequence with the reference. The E29Q, E40K and L93R mutations in E6 protein were also reported in Zhejiang province of China^17^. Due to the limited numbers of HNE02-HNE13 sequences, associations between amino mutation and cervical lesions were not conducted.

For HPV58 L1 gene, five non-synomous mutations, including N82T, L150F, F318Y, I325M and T375N, had been detected in Liaoning province of China^35^. The L150F (15/56) was located in the DE loop of L1 protein, which played an important role in the recognition of VLP^35^. The A6014C, A6416G, T6434C and A6539G in L1 gene variations were present in the whole HPV58 sublineage A2. It was reported that the variations in the fragement of L1 gene (nucleotides 6014–6539) were characterized in HPV58 sublineage A2^35^. In the current research, HPV58 sublineage A1 was the predominant sublineage, which was similar to other provinces of China, such as Zhejiang, Hunan and Liaoning provinces (56.9%)^16,34,35^. In Japan, most HPV58 isolates belonged to lineage A, with more were sublineage A2^40^. Nevertheless, it was reported that there was no association between HPV58 (sub) lineages and cervical lesions^34^. For HPV58 E6 gene, the A388C (K93N) variation was the most predominant mutation (55.4%, 31/56), which have also been reported in Hubei province of China^41^. In our study, no significant difference was observed among HPV58 (K93N) infected females with normal cervix or low-grade lesions. However, it was reported that the K93N can significantly reduce the risk of cervical lesions in Hongkong and Shanghai^42,43^. The most common synomous mutations observed in HPV58 E6 and E7 gene were C307T and T744G, which had also been reported in the past^44–46^. The C307T and T744G mutations were also common in other countries, such as Mexico, Korean and Italy^35,47–49^. However, the variations included in the present study had been reported to have no association with cervical lesions^34,35,49^.

## Conclusion

In summary, the present study provides basic information about the distribution, genotypes and variations of HPV among females population in Luoyang city, which would assist in the formulation of HPV screening and vaccination programs and preventive strategies for HPV-attributable cancer in this region.

## Methods

### Study subjects and specimen collection

From April 2019 to April 2021, 6538 females (rang from 18 to 90 years old, mean, 41.14 ± 11.42) who underwent cervical cancer screening in the 989 Hospital of Joint Service Support Force of Chinese PLA (Luoyang city, Henan province, China) were included in this study. The female was considered if she: (a) had no use of vaginal medication or washing in the previous 72 h; (b) had no sexual activity in the previous 24 h; (c) was not presently during menstruation; (d) had no use of acetic or iodine. Before collection, written informed consent from each participant was obtained. The study protocol adhered to the principles of the Declaration of Helsinki and was approved by the institutional ethics committee (Grant No: LLSC20190305).

### HPV genotyping

Cervical specimens were collected by a gynecological practitioner using a cytobrush from the ecto- and endocervix of uterus. The samples were stored at – 20 °C until the HPV genotyping. The HPV genotype was proceed by a commercial gene chip (Chaozhou Hybribio Limited Corporation, Chaozhou, China) according to the manufacturer’s instruction. The gene chip contained 37 genotype-specific oligonucleotides designed to detect 18 high-risk human papillomavirus (HR-HPV: 16, 18, 26, 31, 33, 35, 39, 45, 51, 52, 53, 56, 58, 59, 66, 68, 73 and 82) and 19 low-risk human papillomavirus (LR-HPV: 6, 11, 34, 40, 42, 43, 44, 54, 55, 57, 61, 67, 69, 70, 71, 72, 81, 83 and 84). The final results were determined by colorimetric change on the chip under direct visualization and blue-purple spots were recognized as HPV positive.

### HPV sequencing

Single HPV18 and HPV58 positive samples were chosen and used to amplify the full length of L1, E6 and E7 genes. The primers were designed based on published HPV 18 (AY262282) and HPV58 (D90400) sequences in GenBank and further synthesized by Sangon Biotech, Inc. (Shanghai, China) (Table 7). Each 50 μl PCR reaction mixture contained 2 μl of each primer, 25 μl 2 × PrimeSTAR Max Premix (Takara Biotechnology Co., LTD, Dalian, China), 19 μl of ultrapure water and 2 μl of template cDNA. The PCR reaction conditions were as follows: 94 °C for 10 min; 35 cycles of 95 °C for 30 s, 60 °C for 30 s, 72 °C for 60 s, 72 °C for 10 min. The amplified products were ligated into p-EASY-Blunt cloning vector (TransGen Biotech, China) according to manufacturer’s instruction and then applied for sequencing by Sangon Biotech, Inc. (Shanghai, China).

**Table 7:** Primers used for the amplification of HPV18 and HPV58 L1, E6 and E7 genes.

Primer name	Start codon	Sequence 5–3	Amplicon size (bp)
HPV18 L1 1F	5258	GTMTCTGCYACGGRGGACAA	1223
HPV18 L1 1R	6480	CACAGCTGCCAGGTGAAGC	
HPV18 L1 2F	6220	CTGGATATGGTGCCAYGGRC	1109
HPV18 L1 2R	7328	CTCACYAGGGCGCAACCACAT	
HPV18 E6E7 F	36	GGAGTRACCRAAAACGGTYG	895
HPV18 E6E7 R	930	CCTTCTGRATCAGCCATTGTT	
HPV58 L1 1F	5600	ATTTTGVGTCGCAGACGTAA	405
HPV58 L1 1R	6004	TGACCACTTACGCCAACACC	
HPV58 L1 2F	5965	TAGGTAGGGGACAGCCATTG	530
HPV58 L1 2R	6494	TGCAGTATTACCGGACCCTT	
HPV58 L1 3F	6424	TTAATAGGGCCGGAAAACTTGG	490
HPV58 L1 3R	6913	GCCTGGGAGGTAACAAATCTAT	
HPV58 L1 4F	6840	GACTGGCAATTTGGTTTAACAC	441
HPV58 L1 4R	7280	CAGGAAACTGACAAAGACATAGA	
HPV58 E6E7 F	42	CGAAAACGGTCTGACCGAAA	968
HPV58 E6E7 R	1009	TATCGTCTGCTGTTTCGTCC	

### Variants and phylogenetic analysis of HPV18 and HPV58

To identify the variations on the HPV18 and HPV58 L1, E6 and E7 genes, the reference HPV18 (GeneBank AY262282) and HPV58 (GeneBank D90400) sequences were selected and compared with the studied sequences. The comparison was proceeding by DNAStar (Madison, WI, USA) and positions of variations were numbered based on the reference sequence.

Phylogenetic trees based on the L1 gene of HPV18 and HPV58 were constructed through Maximum Likelihood method with 1000 bootstrapped replicates using the MEGA (version 6.0). Reference sequences that represent each HPV18 and HPV58 lineage were used to construct the distinct phylogenetic branches.

### Statistical analysis

SPSS version 19.0 (IBM, Armonk, NY, USA) was used to assess the significance of differences in HPV positivity rates among groups. Females with no lesion at colposcopy biopsy were grouped as normal cervices, with CIN2 or worse were grouped as outcome variable. The oncogenic risk association of HPV18 and HPV58 E6/E7 amino acid substitutions was assessed using Chi-squared test or Fisher’s exact test. *P*-value < 0.05 was considered to be statistically significant.

### Ethics approval and consent to participate

Females were informed and a written consent was received. The study protocol adhered to the principles of the Declaration of Helsinki and was approved by the institutional ethics committee in the 989 Hospital of Joint Service Support Force of Chinese PLA, Military Training Medical Research Institute of the Whole Army (Grant No: LLSC20190305).

## Data Availability

The datasets generated during the current study are not publicly available yet, due to privacy concerns and ongoing additional research. Data can be made available for peer review on reasonable request through contacting the corresponding author. The sequences described in manuscript were submitted to GenBank and have gained the accession numbers (OP684017-OP684101).

## Abbreviations

HPV: Human papillomavirus
LR-HPV: Low-risk human papillomavirus
HR-HPV: High-risk human papillomavirus

## References

[1] Sung H (2021). Global Cancer Statistics 2020: GLOBOCAN estimates of incidence and mortality worldwide for 36 cancers in 185 countries. CA Cancer J. Clin..

[2] Ferlay J (2015). Cancer incidence and mortality worldwide: Sources, methods and major patterns in GLOBOCAN 2012. Int. J. Cancer.

[3] Xia C (2022). Cancer statistics in China and United States, 2022: Profiles, trends, and determinants. Chin. Med. J. (Engl.).

[4] Bosch FX, de Sanjose S (2002). Human papillomavirus in cervical cancer. Curr. Oncol. Rep..

[5] Munoz N (2002). Role of parity and human papillomavirus in cervical cancer: The IARC multicentric case-control study. Lancet.

[6] Bosch FX, Lorincz A, Munoz N, Meijer CJ, Shah KV (2002). The causal relation between human papillomavirus and cervical cancer. J. Clin. Pathol..

[7] Bzhalava D, Eklund C, Dillner J (2015). International standardization and classification of human papillomavirus types. Virology.

[8] Munoz N (2003). Epidemiologic classification of human papillomavirus types associated with cervical cancer. N. Engl. J. Med..

[9] de Sanjose S (2010). Human papillomavirus genotype attribution in invasive cervical cancer: A retrospective cross-sectional worldwide study. Lancet Oncol..

[10] Ma L (2019). Characteristics of women infected with human papillomavirus in a tertiary hospital in Beijing China, 2014–2018. BMC Infect. Dis..

[11] Zhang C (2018). Prevalence and genotype distribution of human papillomavirus among females in the suburb of Shanghai, China. J. Med. Virol..

[12] Patel C (2018). The impact of 10 years of human papillomavirus (HPV) vaccination in Australia: What additional disease burden will a nonavalent vaccine prevent?. Euro Surveill..

[13] Kavanagh K (2017). Changes in the prevalence of human papillomavirus following a national bivalent human papillomavirus vaccination programme in Scotland: A 7-year cross-sectional study. Lancet Infect. Dis..

[14] Chen G (2020). Prevalence and genotype distribution of human papillomavirus in women with cervical cancer or cervical intraepithelial neoplasia in Henan province, central China. J. Med. Virol..

[15] Burk RD, Harari A, Chen Z (2013). Human papillomavirus genome variants. Virology.

[16] Zu Y (2021). Genetic characteristics of human papillomavirus type 16, 18, 52 and 58 in southern China. Genomics.

[17] Xu HH (2018). Human papillomavirus (HPV) 18 genetic variants and cervical cancer risk in Taizhou area, China. Gene.

[18] Liu J (2020). Prevalence and genotype distribution of human papillomavirus in Zhengzhou, China, in 2016. Arch. Virol..

[19] Hu S (2021). A nationwide post-marketing survey of knowledge, attitude and practice toward human papillomavirus vaccine in general population: Implications for vaccine roll-out in mainland China. Vaccine.

[20] Ge Y (2019). Prevalence of human papillomavirus infection of 65,613 women in East China. BMC Public Health.

[21] Zhao P (2018). Prevalence and genotype distribution of human papillomavirus infection among women in northeastern Guangdong Province of China. BMC Infect. Dis..

[22] Liu XX (2014). Human papillomavirus prevalence and type-distribution among women in Zhejiang Province, Southeast China: A cross-sectional study. BMC Infect. Dis..

[23] Mai Q, Yang X, Cheng H, Wu G, Wu Z (2020). Prevalence and genotype distribution of human papillomavirus among women with cervical lesions in Shenzhen city, China. Hum. Vaccin. Immunother..

[24] Althoff KN (2009). Correlates of cervicovaginal human papillomavirus detection in perimenopausal women. J. Women's Health.

[25] Duan R, Qiao Y, Clifford G, Zhao F (2020). Cancer burden attributable to human papillomavirus infection by sex, cancer site, age, and geographical area in China. Cancer Med..

[26] Li N, Franceschi S, Howell-Jones R, Snijders PJ, Clifford GM (2011). Human papillomavirus type distribution in 30,848 invasive cervical cancers worldwide: Variation by geographical region, histological type and year of publication. Int. J. Cancer.

[27] Chan PK (2013). Geographical distribution and oncogenic risk association of human papillomavirus type 58 E6 and E7 sequence variations. Int. J. Cancer.

[28] Chan PK (2014). Meta-analysis on prevalence and attribution of human papillomavirus types 52 and 58 in cervical neoplasia worldwide. PLoS ONE.

[29] Tao X (2021). Prevalence and carcinogenic risk of high-risk human papillomavirus subtypes in different cervical cytology: A study of 124,251 cases from the largest academic center in China. J. Am. Soc. Cytopathol..

[30] Serrano B (2015). Human papillomavirus genotype attribution for HPVs 6, 11, 16, 18, 31, 33, 45, 52 and 58 in female anogenital lesions. Eur. J. Cancer.

[31] Xu H (2016). Diagnostic accuracy of high-risk HPV genotyping in women with high-grade cervical lesions: evidence for improving the cervical cancer screening strategy in China. Oncotarget.

[32] Yang R (2005). Papillomavirus capsid mutation to escape dendritic cell-dependent innate immunity in cervical cancer. J. Virol..

[33] Xi LF (2007). Risk for high-grade cervical intraepithelial neoplasia associated with variants of human papillomavirus types 16 and 18. Cancer Epidemiol. Biomark. Prev..

[34] Yu JH (2019). Genetic variability and oncogenic risk association of human papillomavirus type 58 E6 and E7 genes in Taizhou area, China. Gene.

[35] Liu JH (2012). Variations of human papillomavirus type 58 E6, E7, L1 genes and long control region in strains from women with cervical lesions in Liaoning province, China. Infect. Genet. Evol..

[36] Chen AA (2015). Human papillomavirus 18 genetic variation and cervical cancer risk worldwide. J. Virol..

[37] Yamaguchi-Naka M (2020). Molecular epidemiology of human papillomavirus 18 infections in Japanese Women. Infect. Genet. Evol..

[38] Sichero L (2007). High grade cervical lesions are caused preferentially by non-European variants of HPVs 16 and 18. Int. J. Cancer.

[39] Liu H (2019). Cervical human papillomavirus among 19,753 women attending gynecological department of a major comprehensive hospital in north Anhui China 2013–2016: Implication for cervical cancer screening and prevention. J. Med. Virol..

[40] Tenjimbayashi Y (2017). Whole-genome analysis of human papillomavirus genotypes 52 and 58 isolated from Japanese women with cervical intraepithelial neoplasia and invasive cervical cancer. Infect. Agent Cancer.

[41] Yang Z (2022). Genetic variability of E6 and E7 genes of human papillomavirus type 58 in Jingzhou, Hubei Province of central China. Virol. J..

[42] Zhao J (2019). Phylogeny and polymorphism in the E6 and E7 of human papillomavirus: alpha-9 (HPV16, 31, 33, 52, 58), alpha-5 (HPV51), alpha-6 (HPV53, 66), alpha-7 (HPV18, 39, 59, 68) and alpha-10 (HPV6, 44) in women from Shanghai. Infect. Agent. Cancer.

[43] Chan PK (2002). Association of human papillomavirus type 58 variant with the risk of cervical cancer. J. Natl. Cancer Inst..

[44] Liu Y, Pan Y, Gao W, Ke Y, Lu Z (2017). Whole-genome analysis of human papillomavirus types 16, 18, and 58 isolated from cervical precancer and cancer samples in Chinese women. Sci. Rep..

[45] Chen Z (2017). E6 and E7 gene polymorphisms in human papillomavirus types-58 and 33 identified in Southwest China. PLoS ONE.

[46] Yang L (2014). Genetic variability of HPV-58 E6 and E7 genes in Southwest China. Infect. Genet. Evol..

[47] Conde-Ferraez L (2017). Genetic variability in E6 and E7 oncogenes from human papillomavirus type 58 in Mexican women. Intervirology.

[48] Bae JH (2009). Distribution of human papillomavirus type 58 variants in progression of cervical dysplasia in Korean women. J. Microbiol. Biotechnol..

[49] Cento V, Rahmatalla N, Ciccozzi M, Perno CF, Ciotti M (2011). Intratype variations of HPV 31 and 58 in Italian women with abnormal cervical cytology. J. Med. Virol..

